# The management of pemphigus vulgaris and mucous membrane pemphigoid in a joint oral medicine and dermatology clinic: a five-year narrative review

**DOI:** 10.1038/s41415-024-7074-8

**Published:** 2024-02-23

**Authors:** Gemma Davis, Russell Hathway, Debbie Shipley, Konrad Staines

**Affiliations:** 4141524584001 Specialty Registrar and Honorary Clinical Lecturer, Department of Oral Medicine, Bristol Dental Hospital, Lower Maudlin Street, Bristol, BS1 2LY, UK; 4141524584002 Consultant Dermatologist and Honorary Senior Clinical Lecturer, Department of Dermatology, Bristol Royal Infirmary, 1 Marlborough Hill Pl, Bristol, BS2 8HA, UK; 4141524584003 Consultant and Honorary Professor in Oral Medicine, Department of Oral Medicine, Bristol Dental Hospital, Lower Maudlin Street, Bristol, BS1 2LY, UK; Bristol Dental School, 1 Trinity Quay, Avon Street, Bristol, BS2 OPT, UK

## Abstract

Pemphigus disease and mucous membrane pemphigoid are autoimmune blistering diseases (AIBDs) which may involve both oral and extra-oral tissues. The Bristol Joint Oral Medicine and Dermatology Combined Clinic was set up in 2014, with the primary aim of improving the standard of care for patients with AIBDs. This interdisciplinary approach aimed to address the medical management challenges due to the multisite nature of these AIBDs.

We present a narrative report of the clinical work undertaken within this clinic, focused on the management of this patient cohort within a five-year span (2017-2022). This report outlines the multisite nature of AIBDs and the range of topical and systemic treatments that were employed to achieve adequate disease control and optimise outcomes for patients. We reflect on the experiential benefits of this multidisciplinary clinic extended beyond immediate patient benefits to areas such as specialist training, both from a dermatologist's and oral physician's perspective.

## Key points

- Improve knowledge of autoimmune blistering diseases relevant for the general dental practitioner.
- Improve awareness of the varied clinical presentation of autoimmune blistering diseases relevant for a general dental practitioner.
- Reinforce the benefits of multidisciplinary teamworking for both patients and clinicians in the management of autoimmune blistering diseases.

## Introduction

Pemphigus and pemphigoid diseases (PD/PGD) are autoimmune blistering diseases (AIBDs), with potential involvement of cutaneous and/or mucosal tissues. The underlying pathophysiology of PD involves autoantibodies targeting structural antigens at keratinocyte-to-keratinocyte junctions, whilst in PGD, these autoantibodies target basement zone antigens, resulting in intraepithelial and subepithelial blisters, respectively.^1^^,^^2^^,^^3^^,^^4^

PDs encompasses three main conditions: pemphigus vulgaris (PV), pemphigus foliaceus (PF) and paraneoplastic pemphigus (PNP). PV is the most commonly encountered type in oral medicine clinical practice, as PF rarely involves oral tissues and PNP is extremely rare.^5^In most cases of PV, the oral mucosa is the first site of presentation with extension to involve the skin occurring later in the disease process in some cases.^6^^,^^7^

PGD is a group of eight autoimmune disorders.^3^ Bullous pemphigoid (BP) is the most common form that typically involves the skin with minimal long-term mucosal involvement. Mucous membrane pemphigoid (MMP) on the other hand is often seen within oral medicine clinics, consequent to the significant mucous membrane involvement including the oral cavity (85%), conjunctiva (65%), nasal cavity (20-40%), anogenital area (20%), pharynx (20%), larynx (5-10%) and oesophagus (5-15%). Skin involvement in MMP is mild and is seen in 25-30% of patients.^3^^,^^4^

## Pemphigus vulgaris

### Epidemiology

Robust epidemiological data for PV are limited. In the UK, the reported incidence is 0.68 cases per 100,000 people per year.^8^ The incidence varies widely between ethnic groups, with individuals of Middle Eastern and Jewish descent more likely to be affected compared to those from Western Europe.^9^ The fifth and sixth decades of life are the most prevalent onset years with both sexes equally affected.^10^ PV is associated with an increase in overall mortality, mainly related to the immunosuppressive treatment required with respiratory tract infections and septicaemia often reported as causes of death.^11^^,^^12^

### Clinical presentation

**Fig. 1:**
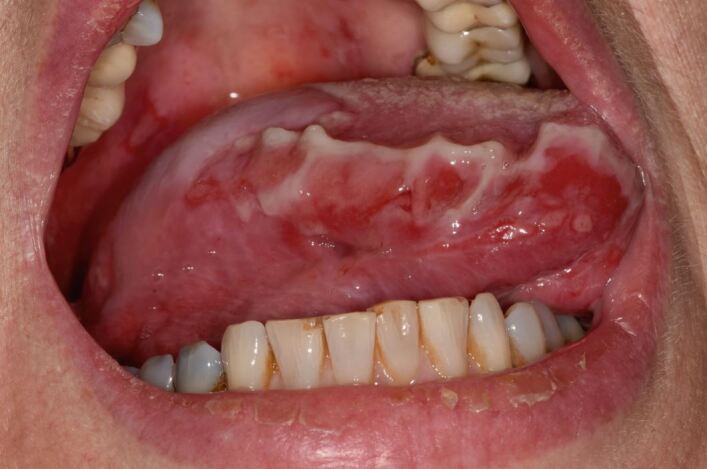
Erosions on right lateral border of tongue in PV

**Fig. 2:**
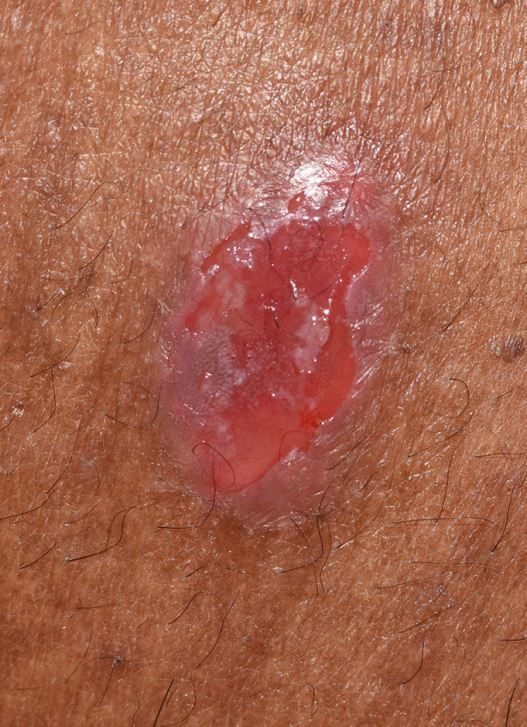
Ruptured cutaneous blister in PV

PV may involve one or more epithelial tissues, most commonly the oral mucosa (Fig. 1) and skin (Fig. 2). Involvement of the conjunctival, laryngeal, oesophageal, genital and nasal mucosae may also occur, albeit less frequently.^7^ The oral presentation of PV is characterised by flaccid blisters and persistent painful erosions typically presenting on the buccal mucosa and/or gingivae.^13^

### Aetiopathogenesis

The aetiopathogenesis of PV is complex and the exact mechanism is currently unknown. It is proposed that an environmental trigger in a genetically predisposed individual results in the formation of autoantibodies directed towards desmosomal proteins, primarily desmoglein 1 (Dsg1) and desmoglein 3 (Dsg3). This results in intraepithelial blister formation secondary to acantholysis; separation of epithelial keratinocytes from each other.^14^

### Diagnosis

The diagnosis of PV requires clinicopathological correlation and is based on four criteria. These are clinical presentation, histopathology, direct immunofluorescence (DIF) microscopy and indirect immunofluorescence (IIF).^13^ PV is characterised histologically by intraepithelial blister formation and immunofluorescence studies showing immunoglobulin G (IgG), immunoglobulin A (IgA) and/or complement component 3 (C3) binding on the cell surfaces of keratinocytes. Newer immunological techniques such as enzyme-linked immunosorbent assays (ELISAs) can be used to detect specific anti-Dsg1 and anti-Dsg3 autoantibodies.^6^

### Treatment

Immunosuppressive therapy to induce remission and thereafter maintain disease control constitutes the typical systemic PV treatment protocol.^15^ Induction is achieved using systemic corticosteroids and disease control maintained using adjuvant systemic immunosuppressant drugs such as azathioprine (AZA) or mycophenolate mofetil (MMF).^7^ In individuals with refractory or recurring disease, newer anti-CD20 agents, such as rituximab (RTX) and intravenous immunoglobulin therapy (IVIg) may be used.^13^ RTX is currently approved by NHS England (NHSE) as a third-line treatment for PV and it is estimated that approximately 1% of patients will require this treatment.^16^

## Mucous membrane pemphigoid

### Epidemiology

MMP is a rare disease with an estimated incidence of 1-2 new cases per million people each year in Germany and France.^17^^,^^18^ The fifth and sixth decades of life are the most prevalent onset years for MMP, with the disease occurring almost twice as frequently in women than men.^10^

### Clinical presentation

**Fig. 3:**
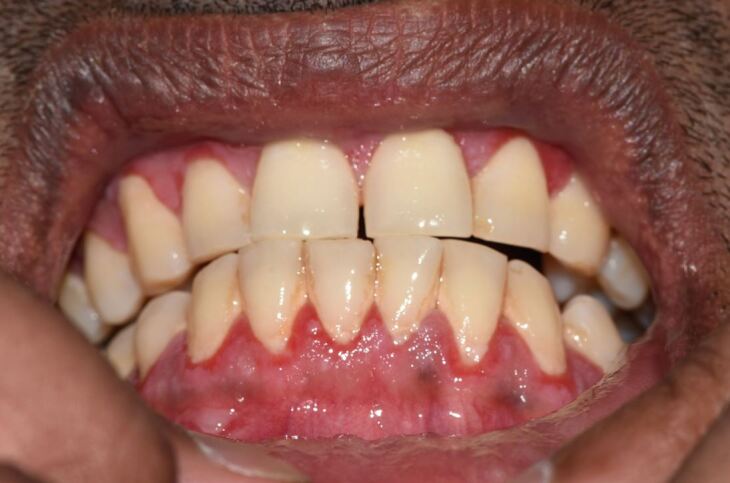
Desquamative gingivitis, as observed in MMP

**Fig. 4:**
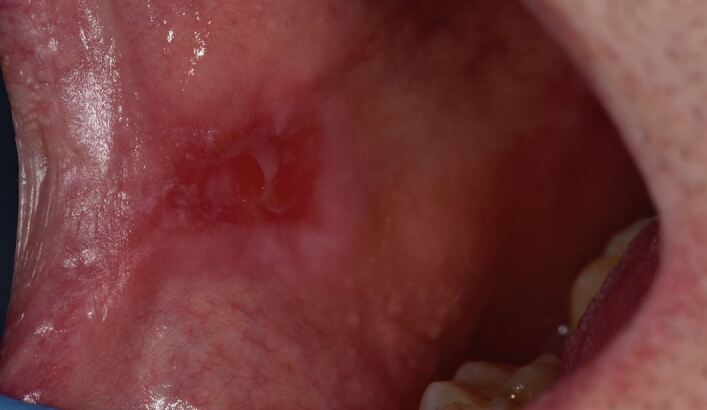
Ruptured oral blister on right commissure, as observed in MMP

**Fig. 5:**
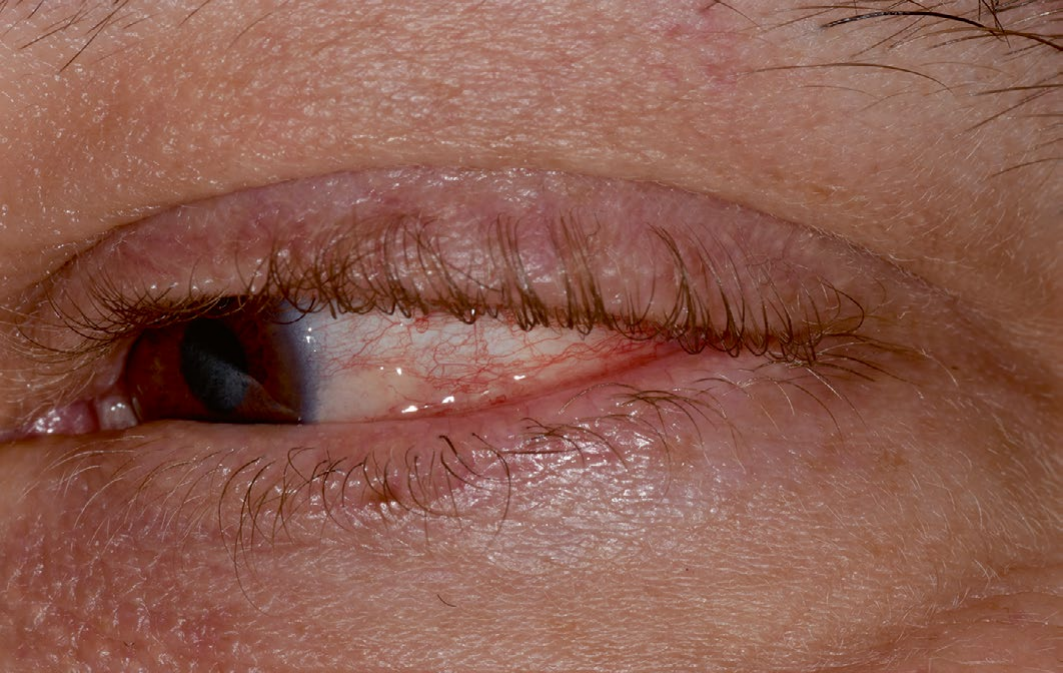
Conjunctivitis and early symblepharon formation in left eye, an ocular manifestation of MMP

MMP affects one or more mucosal sites, including the oral, ocular, laryngeal, pharyngeal and anogenital mucosa. The skin is affected in 20-35% of MMP cases but is typically only mild. When involved, skin lesions tend to more frequently involve the head, neck and upper body; however, more generalised skin lesions have been reported.^2^ The severity of MMP ranges from mild disease with minimal symptoms to severe multisite disease with significant pain, blistering and scarring.^19^ The oral lesions in MMP may be indistinguishable clinically from PV with a similar picture of blisters, erosions and a desquamative gingivitis (Fig. 3, Fig. 4).^20^ The potential for scarring is a feature of MMP, not typically encountered in PV, which can result in irreversible complications, such as symblepharon in ocular pemphigoid (Fig. 5) and airway compromise from laryngeal stenosis.^10^^,^^21^

### Aetiopathogenesis

As for PV, the exact aetiopathogenesis of MMP is currently poorly understood. It is hypothesised that a genetic predisposition, along with an environmental trigger, results in the production of autoantibodies directed against several epithelial basement membrane antigens.^22^ To date, six autoantigens have been identified: BP180 (bullous pemphigoid antigen 2), BP230 (bullous pemphigoid antigen 1), laminin 332, collagen XII and both subunits of the α6β4 integrin.^23^

### Diagnosis

The diagnosis of MMP requires clinicopathological correlation using the same four criteria as PV.^19^ MMP is characterised histologically by subepithelial blister formation and by linear deposition of IgG, IgA and/or C3 along the basement membrane zone on immunofluorescence microscopy. Detection of circulating autoantibodies directed against BP180 and BP230 can be detected using ELISAs.

### Treatment

Topical steroids may be sufficient as a first-line treatment in mild/moderate MMP confined to oral mucosal tissues, with systemic medication reserved for more severe or multisite disease. Examples of first-line systemic therapies for mild/moderate MMP include dapsone and tetracyclines.^19^ For more severe MMP, immunosuppressive therapies, including systemic corticosteroids, MMF and AZA, can be used.^19^^,^^22^ As for PV, RTX is considered in the treatment of MMP should conventional therapies fail to control the disease. RTX is currently commissioned by NHSE as a fourth-line treatment option in MMP.^16^

## The Bristol Joint Oral Medicine and Dermatology Combined Clinic

The care for patients with multisite AIBDs requires input from an oral physician, dermatologist, otolaryngologist and ophthalmologist to combine expertise in order to institute appropriate therapy and improve outcomes.^10^^,^^23^ The Bristol Joint Oral Medicine and Dermatology Combined Clinic (BJOMDCC) was set up in 2014, in recognition of the above, to support the delivery of enhanced integrated care. The clinic receives referrals of patients from a tertiary catchment area of approximately seven million patients. The BJOMDCC is a half-day clinic held once a month and consists of a consultant dermatologist, a consultant oral physician and specialty trainees from both dermatology and oral medicine.

This article presents the demographic data, disease features, investigations performed and treatments used for the cohort of patients with a diagnosis of PV or MMP who attended the BJOMDCC from January 2017 until December 2022.

### Methods

Patients seen in the BJOMDCC between January 2017 and December 2022 were identified using the trust's CareFlow electronic patient record database *(*System C, UK*).* A manual search of these patients identified those with a diagnosis of PV or MMP. Data on patient demographics, intra-oral and extra-oral disease features, diagnostic investigations performed and their results, and treatments used were collected and recorded in a digital proforma on Microsoft Excel.

### Results

#### Patient demographics

A total of 14 cases of PV (six men, eight women) and 25 cases of MMP (9 men, 16 women) were identified. The mean age at time of initial presentation for PV was 64 years (range 39-83) and for MMP was 69 years (range 44-99). The average follow-up duration for PV was 36 months and MMP was 28 months, with a mixture of face-to-face and remote consultations which we adopted during the COVID pandemic.

#### Clinical features

The intra-oral and extra-oral sites of involvement for PV and MMP are described below.

##### PV

The most common oral site of involvement in PV was the buccal mucosa (n = 14; 100%), followed by the tongue (n = 11; 79%), soft palate (n = 7; 50%), lip vermillion (n = 3; 21%), gingiva (n = 2; 14%) and labial mucosa (n = 1; 7%).

Extra-oral sites of involvement included skin (n = 11; 79%), anogenital (n = 3; 21%), pharyngeal (n = 2; 14%), nasal (n = 2; 14%), laryngeal (n = 1; 7%) and ocular (n = 1; 7%) mucosa.

##### MMP

The most common oral site of involvement in MMP was the gingiva (n = 22; 88%), followed by the buccal mucosa (n = 12; 48%), soft palate (n = 11; 44%), hard palate (n = 3; 12%), tongue (n = 1; 4%), lip vermillion (n = 1; 4%) and floor of mouth (n = 1; 4%).

Extra-oral sites of involvement included skin (n = 22; 88%), pharyngeal (n = 14; 44%), anogenital (n = 10; 40%), laryngeal (n = 9; 36%), ocular (n = 7; 28%), and nasal (n = 2; 8%) mucosa.

#### Investigations

Diagnostic tests were undertaken in all patients (n = 39; 100%) and are summarised in Table 1.

**Table 1:** Investigations utilised in the diagnosis of AIBDs

**Diagnostic investigation**	**Percentage completed: PV**	**Percentage completed: MP**	**Diagnostic yield: PV (%)**	**Diagnostic yield: MMP (%)**
Histopathology	100	100	93	96
Direct immunofluorescence	100	100	86	76
Indirect immunofluorescence	86	68	100	12
ELISA	14	0	100	0

##### PV

An oral biopsy submitted for histopathology was performed in all patients (n = 14) with supportive histological features of PV seen in 93% (n = 13). All cases (n = 14) had a perilesional oral biopsy for DIF, of which 86% (n = 12) were consistent with PV. Additionally, 86% of patients (n = 12) had serum collected for IIF and all (n = 12) were consistent with a diagnosis of PV. ELISA studies for Dsg1 and Dsg3 were performed in two cases (14%), with both returning positive results.

##### MMP

An oral biopsy was performed in all patients (n = 25) with supportive histological features of MMP seen in 96% (n = 24). All cases (n = 25) had a perilesional biopsy for DIF, of which 76% (n = 19) were consistent with MMP. A total of 17 patients (68%) had serum collected for IIF, with three (18%) reports supportive of a diagnosis of MMP. No ELISAs for BP180 and BP230 were performed.

#### Treatment

The use of topical corticosteroid therapy was recorded in all patients with a diagnosis of PV and MMP (n = 39). The most common topical agents used were betamethasone sodium phosphate 500 microgram soluble tablets made into a mouthwash used up to four times daily, and fluocinolone acetonide 0.025% gel applied up to twice daily.

**Fig. 6:**
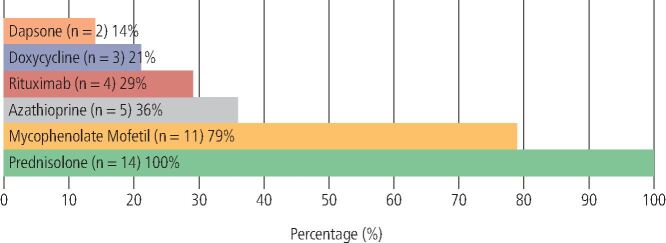
Therapeutic agents used in the management of PV (n = 14)

**Fig. 7:**
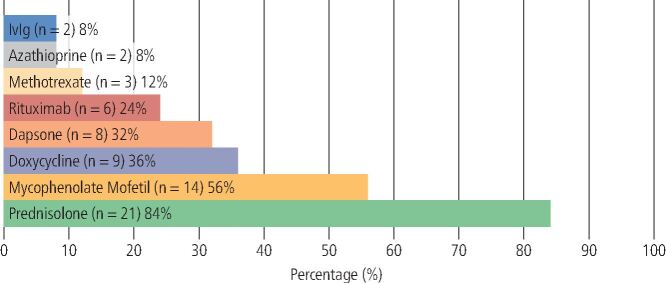
Therapeutic agents used in the management of MMP (n = 25)

Systemic therapy was prescribed for all patients (n = 39). The range of systemic medications used is outlined in Figure 6 (PV) and Figure 7 (MMP).

##### PV

The most prescribed systemic agent in PV was prednisolone (n = 14; 100%), followed by MMF (n = 11; 79%), AZA (n = 5; 36%), RTX (n = 4; 29%), doxycycline (n = 3; 21%) and dapsone (n = 2; 14%).

Layering of medications with two or more concomitantly prescribed immunomodulant or immunosuppressant agents was recorded in 93% (n = 13) of patients. The most used combination of systemic agents was prednisolone and MMF.

##### MMP

The most prescribed systemic agent in MMP was prednisolone (n = 21; 84%), followed by MMF (n = 14; 56%), doxycycline (n = 9; 36%), dapsone (n = 8; 32%), RTX (n = 6; 24%), methotrexate (n = 3, 12%), AZA (n = 2; 8%) and IVIg (n = 2; 8%). Additionally, 88% (n = 22) of MMP patients were co-prescribed two or more systemic therapies, with the most common combination being prednisolone coupled with MMF.

#### Adverse effects of disease and treatment

##### PV

There were no recorded irreversible complications of disease associated with PV; however, one patient required hospitalisation for supportive care following an acute flare-up restricting oral intake.

The most common adverse effects of systemic therapy were gastrointestinal upset (n = 2; 14%), anaemia (n = 1; 7%), neutropenia (n = 1; 7%), AZA-induced hepatotoxicity (n=1; 7%) and RTX-induced angioedema (n = 1; 7%).

##### MMP

Recorded irreversible complications of disease associated with MMP included pharyngeal scarring without stenosis (n=4; 16%), pharyngeal scarring with supraglottic stenosis requiring permanent tracheostomy (n = 1; 4%) and conjunctival scarring (n = 2; 8%).

Adverse effects of systemic therapy used for MMP included the development of non-melanoma skin cancer (n = 2; 8%), bone marrow suppression (n = 2; 8%), AZA-induced hepatotoxicity (n = 1; 4%) and MMF-related insomnia (n = 1; 4%).

### Discussion

PV and MMP are AIBDs characterised by circulating autoantibodies directed at epithelial antigens.^24^ The classification and management of these diseases is based on clinical, histological, direct and indirect immunofluorescence findings.

The distribution of disease involvement for PV and MMP in this cohort is consistent with other reports in the literature.^25^

DIF remains the gold standard investigation to identify relevant autoantibody binding in PV and MMP. The sensitivity of IIF is generally lower than DIF, especially in MMP, owing to the lower titre of circulating autoantibodies.^19^ This was demonstrated in our cohort, with only 18% of MMP cases returning a true positive IIF result. However, it is our opinion that this is still a valuable investigation that can aid diagnosis and monitor disease activity when performed serially.

We recognise that the use of ELISA testing for Dsg1, Dsg3, BP180 and BP230 was limited in our patient cohort. This reflects the lack of current universal availability within secondary and tertiary care services nationally. Currently, these investigations are performed at an external laboratory; however, as the BJOMDCC continues to expand, we hope that this will justify adoption of these techniques by our local immunology laboratory, thereby supporting more routine use.

The aim of treatment for AIBDs is targeted at inflammation control and reduction of complications secondary to the disease process. For example, in MMP, uncontrolled inflammation can lead to scarring of extra-oral tissues, such as the conjunctiva, resulting in vision loss.^10^^,^^23^ Unlike extra-oral sites, scarring is rarely seen in the oral cavity due to the rapid epithelial turnover.^2^ However, uncontrolled intra-oral disease impacts on nutrition, oral hygiene, quality of life, and has psychosocial implications. Inflammation and discomfort within the oral cavity can be a barrier towards effective oral hygiene measures, with potential risks of caries development and periodontal attachment loss secondary to poor oral hygiene. Due to significant time pressures within the BJOMDCC, we have not been able to routinely collect quality of life metrics or oral disease severity scores which can be helpful in monitoring disease activity and response to treatment. As part of a service re-evaluation, we are looking at re-designing the clinic templates to support collection of this information.

- Learning point: patients with AIBDs may have to contend with a painful desquamative gingivitis hindering their oral hygiene. These patients require regular professional mechanical plaque removal performed by their general dental practitioner to ensure their dental and periodontal health is maintained.

Topical steroid therapy is considered first-line treatment for oral mucosal involvement in AIBDs. With significant disease activity, control of inflammation may not be achievable with topical treatments alone. In such cases, a stepladder approach with the addition of systemic steroid therapy in combination with one or more immunomodulatory or immunosuppressant medications is recommended by current guidelines.^7^^,^^23^

Conventional systemic immunosuppression remains the mainstay approach within the BJOMDCC, reflecting the severity and multisite involvement in this cohort. Combination therapy with two or more immunomodulant or immunosuppressant medications was identified in 93% and 88% of patients with PV and MMP, respectively. This approach allows use of lower doses of individual medications, reducing potential adverse effects, and is consistent with similar approaches within the literature.^13^^,^^19^^,^^26^

The therapeutic landscape has evolved significantly over the last decade with the development of monoclonal antibody therapies.^1^ RTX is a chimeric murine-human monoclonal antibody directed against CD20 positive B-lymphocytes^7^ which works by reducing the number of circulating B-cells and prevents their maturation into antibody-secreting plasma cells. In our cohort, RTX was used as part of the treatment regime for 29% and 24% of patients with PV and MMP, respectively. There is currently a wide variation of access to and uptake of biologic therapy for AIBDs within the UK. Challenges include variation in clinicians' readiness to use newer drugs and local factors, such as availability of biologics multidisciplinary clinics that are not always directly available to oral medicine specialists. Through the BJOMDCC, we have been able to implement Getting it Right First Time recommendations to address variation in uptake and use of biological medicines to ensure patients have equitable access to appropriate therapies.^27^

- Learning point: whilst conventional immunosuppressive agents are still the mainstay of systemic treatment, as more selective biological therapies are developed with less significant risk profiles, it is likely that these drugs will be used earlier and for milder disease.

### Reflections on our experience of this combined clinic

The BJOMDCC was established in 2014 in recognition of the need for a multidisciplinary approach. Our aims were to enhance the quality of care provided, improve outcomes and improve patient experience. This approach has proven popular with patients who benefit from the opportunity to engage in shared care decision-making with key clinicians during a single visit.

Combining our expertise in a collegiate manner has cultivated an environment whereby professional development can flourish, with additional training opportunities for both oral medicine and dermatology specialty trainees alike. We consider this joint clinic approach should be complementary to, rather than replace, conventional single consultant outpatient management. Our experience has been that in addition to managing multisite disease, a joint clinic approach has been helpful in making critical decisions around treatment approaches in patients with single site disease who may have complex medical co-morbidities and immunosuppression requirements.

Initial barriers to developing this combined clinic included the costs involved in two consultants seeing the same patients, scheduling of clinics (with regard to individual job plans) and organisational management of appointments. These obstacles, albeit challenging, were overcome, helped by evidence that the BJOMDCC reduced patient burden with a reduction in the number of appointments required. These costs remain a barrier to expanding the specialist input through direct presence in the clinic of other relevant specialties, such as otolaryngology. This aligns with evidence from similar clinics and is of particular significance, as several of our patients travel significant distances to access their care.^28^

## Conclusion

MMP and PV both have the potential to adversely impact quality of life and result in significant morbidity. These diseases may involve multiple sites; hence, pooling expertise in a multidisciplinary clinic facilitating appropriate management is paramount in providing high-quality holistic care.

Managing complex multisite disease within a combined clinic is effective and has wide-ranging benefits for both patients and clinicians. The BJOMDCC facilitates discussion between physicians, with exploration of potential management strategies from different viewpoints, thereby avoiding ‘care silos' with the risk that this brings when each specialist focusses on their own clinical priorities without joined-up thinking.

Setting up combined clinics can be challenging in the short term; however, this should not deter organisations and clinicians from pursuing such an approach when tangible patient benefit can be achieved.^27^
